# Inhomogeneous myocardial stress perfusion in SPECT studies predicts future allograft dysfunction in heart transplant recipients

**DOI:** 10.1186/s13550-015-0129-8

**Published:** 2015-10-05

**Authors:** Christian Wenning, Alexis Vrachimis, Angelo Dell´Aquila, Alvyda Penning, Jörg Stypmann, Michael Schäfers

**Affiliations:** Department of Nuclear Medicine, University Hospital Münster, Albert-Schweitzer-Campus 1, Building A1, 48149 Münster, Germany; Department of Cardiovascular Medicine, University Hospital Münster, Münster, Germany; Department of Thoracic and Cardiovascular Surgery, University Hospital Münster, Münster, Germany; European Institute for Molecular Imaging—EIMI, University of Münster, Münster, Germany; DFG Cluster of Excellence EXC 1003 ‘Cells in Motion’, University of Münster, Münster, Germany

**Keywords:** Heart transplantation, Gated perfusion SPECT, Inhomogeneous myocardial perfusion, Cardiac allograft vasculopathy, Ejection fraction

## Abstract

**Background:**

Myocardial perfusion gated single photon emission computed tomography (SPECT) can be used for non-invasive detection of coronary artery stenosis and cardiac allograft vasculopathy (CAV), which is a crucial factor for the long-term survival of heart transplant (HTx) recipients. A frequently observed finding in myocardial perfusion imaging of patients after HTx is inhomogeneous myocardial perfusion. This finding is not associated with epicardial CAV, but its prognostic relevance is unclear so far. We therefore evaluated the prognosis of patients with homogeneous versus inhomogeneous myocardial stress perfusion.

**Methods:**

One hundred four HTx patients (mean 3.6 ± 2.9 years after HTx) without significant stress-induced ischemia (summed stress score ≤3) in gated SPECT and without CAV were included. Myocardial stress perfusion was visually assessed as homogeneous, moderately, or severely inhomogeneous. The mean follow-up period after SPECT was 9.4 ± 3.1 years. End points were the diagnosis of CAV, major cardiac events (MACE) or death, and the development of allograft dysfunction (left ventricular ejection fraction, LVEF <45 %).

**Results:**

Of all HTx patients, 24 % enrolled in this study (*n* = 25) presented with inhomogeneous myocardial perfusion. Compared to the patients with homogeneous perfusion, these patients were at higher risk for developing allograft dysfunction (multivariate hazard ratio, HR = 5.59). As to the development of CAV, the occurrence of MACE, or death, no statistical differences were observed between patients with homogenous and inhomogeneous perfusion. There was no correlation between myocardial perfusion pattern and prior cardiac allograft rejections.

**Conclusions:**

Inhomogeneous myocardial stress perfusion in SPECT studies predicts a higher risk for future development of allograft dysfunction in HTx patients (LVEF <45 %) but is not associated with future CAV, MACE, or overall survival.

## Background

Cardiac allograft vasculopathy (CAV) is a major complication associated with decreased long-term survival in heart transplant recipients [1, 2]. CAV is a primary immune-mediated process which differs from other vasculopathies such as coronary artery disease (CAD). The pathophysiological process in the vessel wall in CAV patients is characterized by a diffuse concentric intimal proliferation affecting both the epicardial and the endocardial arteries with no preferential localization [3, 4]. In the course of the disease progressive heart failure, arrhythmia and sudden cardiac death may occur [1]. Once severe CAV has developed, retransplantation is the only definitive treatment option [5]. Serial coronary angiography combined with intravascular ultrasound (IVUS) is the clinical standard for the detection of significant CAV [6]. However, non-invasive myocardial perfusion imaging (MPI) is increasingly gaining attention as an attractive alternative monitoring tool for clinically significant CAV, providing information on both the perfusion provided by the main epicardial vessels and the microcirculation [7]. Although the sensitivity of MPI for the detection of CAV is reported to be lower than for the detection of CAD [6], several clinical studies have identified perfusion deficits assessed by MPI as strong predictors for cardiac events in patients after heart transplantation (HTx) [7–9]. Recent results indicate that stress-induced ischemia and left ventricular dysfunction in HTx patients assessed by gated single photon emission computed tomography (SPECT) correlate with prognosis [10, 11].

Apart from the presence of ischemia or scarring, a frequent finding in MPI of heart transplant recipients is inhomogeneous myocardial perfusion in angiographically normal patients [12]. Previous studies showed that inhomogeneous myocardial perfusion is rare within the first year after transplantation but more frequently observed over time [13, 14]. In a mixed cohort also including patients with known CAV, the prevalence was up to 38 % [11]. Interestingly, the finding is not associated with the prevalence of CAV in angiography at the time of imaging [13, 15]. Even when performed with IVUS, coronary angiography did not detect any kind of CAV in patients presenting with inhomogeneous myocardial perfusion pattern [15]. Thus, perfusion inhomogeneity is assumed to reflect microvasculopathy which is not detectable by angiography [12, 15], although this hypothesis has not been proven so far. Despite these correlative findings, the prognostic relevance of inhomogeneneity in MPI of heart transplant recipients remains unclear. Therefore, the present study evaluates the prognostic impact of inhomogeneous myocardial perfusion in heart transplant recipients without CAV in coronary angiography.

## Methods

### Study population

One hundred sixty-one HTx recipients (137 males, 24 females, mean age at HTx 50.7 ± 12.2 years) undergoing orthotopic cardiac allograft transplantation between 1990 and 2004, who underwent myocardial perfusion gated SPECT followed by coronary angiography on a routine basis between 04/2000 and 01/2006 at the University Hospital Münster were identified. Patients with significant myocardial ischemia and/or scar (summed stress score (SSS) ≥4; *n* = 42), evidence of significant epicardial CAV in coronary angiography (> 50 % stenosis; *n* = 33), and/or left ventricular ejection fraction (LVEF) <45 % (*n* = 25) at the start of the study were excluded. A time interval of 4 weeks between coronary angiography and the SPECT study was tolerated. This resulted in a total population of 104 patients.

The institutional review board of the Medical Association of Westfalen-Lippe and the Faculty of Medicine, University of Münster approved this retrospective study. For the calculation of a threshold for LVEF data from an age-matched, site-specific control group of 80 patients with normal perfusion, a low pre-test likelihood of CAD and without prior cardiac interventions (50 men, 30 women, mean age 50.5 ± 9.6 years; *p* = not statistically significant versus HTx patients) was used. The threshold for normal LVEF was defined as the mean value ± two standard deviations.

### Gated SPECT and image analysis

In all patients, gated SPECT was performed with ^99m^Tc-tetrofosmin as the perfusion agent (MYOVIEW™, General Electric Company, Fairfield, CT, USA). Each vial was reconstituted with ^99m^Tc-O4- according to the manufacturer’s instructions.

All investigations were performed by a sequential stress/rest study (1-day protocol). The stress test was performed according to international standards by physical exercise. The assessment of cardiac function was based on gated SPECT data obtained under resting conditions. For the stress study, a standard dose of about 250 MBq^99m^Tc-tetrofosmin was injected at peak stress while about 750 MBq were administered under resting conditions. All patients received their standard prescribed medication only directly after the stress test, whereas they did not take their medication 12 h prior to the stress test.

Data acquisition started 30 to 60 min after intravenous tracer injection (ECAM, Siemens Gammasonics; 2 heads, 24 projections, 64 × 64 matrix, 180° orbit, 50 s per step, 8 ECG-gates, zoom factor = 1.45). Flood sources were used to obtain transmission data for attenuation correction. Data were reconstructed into static data sets with and without attenuation correction for perfusion analysis and, for the rest study, into ECG-gated data sets for functional analysis (filtered backprojection, butterworth filter, cutoff 0.6, order 5). Reconstructed images were resliced into short axis, horizontal long axis, and vertical long axis for clinical reading. Tracer uptake was semiquantitatively scored for ischemia/scarring from three representative short axis slices (apical, mid-ventricular and basal) and a long axis slice using the 17-segment model. Each of the 17 segments was scored by two independent and experienced observers blinded to the angiographic results according to the ASNC guideline for semiquantitative analysis [16]. Accordingly, summed rest score (SRS), SSS, and summed difference score (SDS) were calculated.

**Fig. 1:**
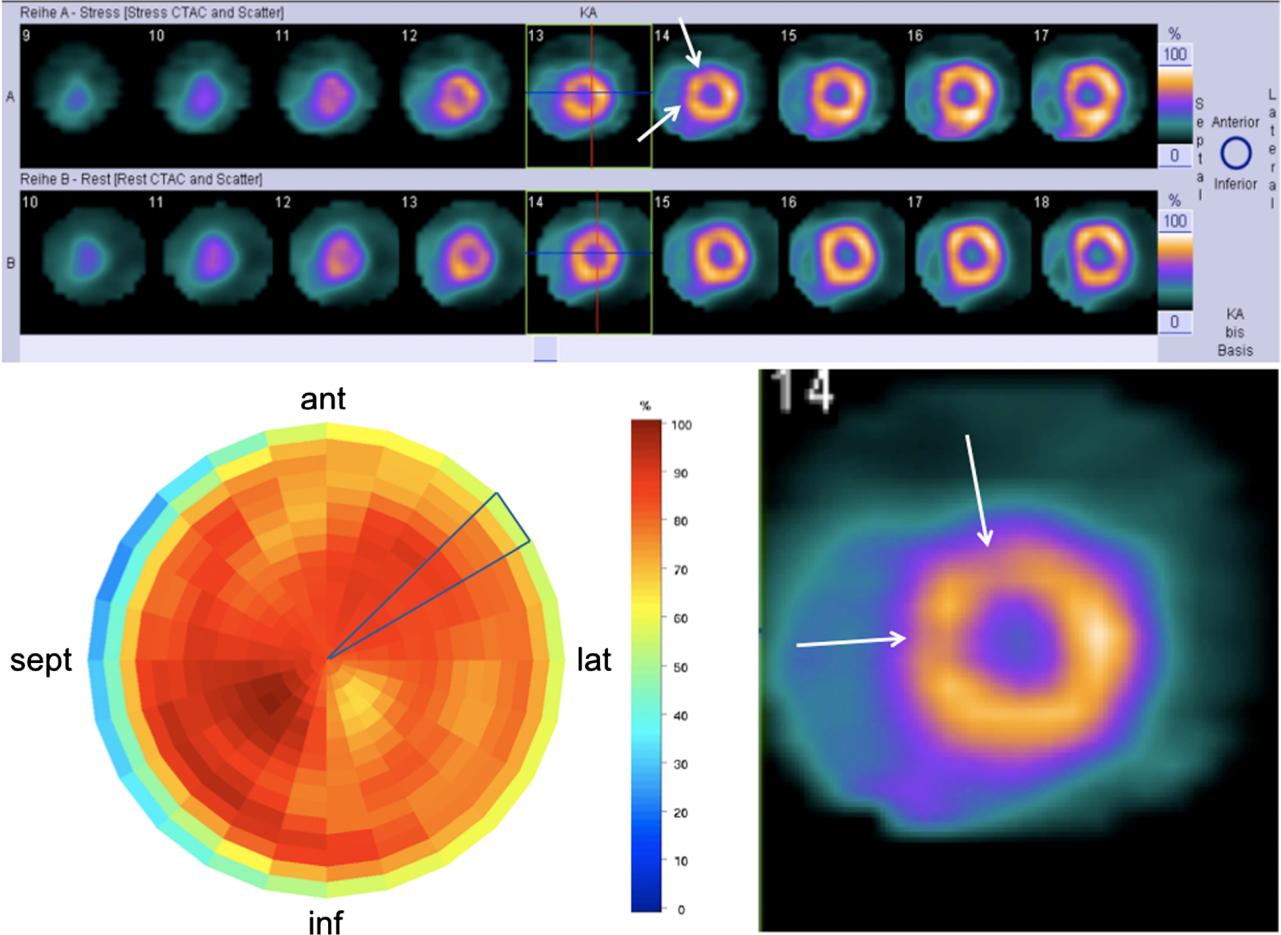
Short axis view of a patient presenting with inhomogeneous perfusion pattern in the stress study (*upper and lower right panel*). Small subsegmental areas of perfusion impairment are marked with *white arrows*. Bull’s eye plot of the left ventricle for analysis of inhomogeneity (*lower left panel*) showing 384 subsegments arranged in 24 sectors (15°). One sector is exemplarily diagrammed

Left ventricular endsystolic and enddiastolic volumes (ESV, EDV) and LVEF were calculated from the ECG-gated SPECT data using a validated contour finding algorithm (ESM, elastic surface model) [17]. Left ventricular myocardium was segmented into 24 sectors, each 15° wide and containing 16 small segments arranged from the apex to the basis of each sector, resulting in 384 segments (Fig. 1). In each case, the three outer, most basal segments (*n* = 72) were excluded from analysis to exclude segments with physiologically decreased uptake in the membraneous septum. This resulted in a total of 312 small segments to analyze. On that basis, myocardial stress and rest perfusion was then graded into 3° of inhomogeneity (no/minimal, moderate, and severe) by two experienced observers, blinded to the results of the clinical follow-up as follows: segments showing a tracer uptake of less than 70 % of the maximum myocardial uptake were considered as pathological. Cases presenting with <10 % pathologic segments were graded as homogeneous, cases with 10–20 % pathological segments were graded as moderately inhomogeneous, and cases showing a decreased uptake in > 20 % of the segments were considered as severely inhomogeneous. Pathological segments did not need to be adjacent. Since no other reliable reference standard for the grading of myocardial perfusion inhomogeneity exists so far, visual grading performed by two independent observers was used as reference. The two observers experienced in nuclear cardiology and blinded towards the clinical data independently graded the degree of inhomogeneity. The kappa values for the intra- and interobserver agreement were 0.911 and 0.858, respectively. In single cases of disagreement between the observers, a consensus was found for final grading.

### Coronary angiography

Coronary angiography was performed using the Judkins technique [18]. Angiograms were analyzed visually by a consensus of two experienced observers blinded to the patient’s medical history and to the SPECT results. As mentioned above, patients with angiographical findings of CAV [3] were excluded from the analysis.

### Echocardiography

Routine transthoracic echocardiograms (TTE) were performed every 3 months from the time of SPECT imaging by experienced cardiac sonographers according to the guidelines of the American Society of Echocardiography (ASE) [19]. LVEF was assessed in every patient.

### Follow-up

Assessment of the left ventricular function in the follow-up was performed by TTE every 3 months. Serial protocol coronary angiography was performed once a year to assess the presence of epicardial CAV.

End points were defined as: manifestation of CAV in coronary angiography, major cardiac event (MACE: coronary revascularization, myocardial infarction, cardiac death or re-transplantation), death from any cause, and the development of allograft dysfunction (LVEF <45 %) assessed by TTE.

### Statistical analysis

Statistical analysis was performed using the SPSS software package (IBM SPSS Statistics for Mac, Version 22.0. Armonk, NY: IBM Corp.). Analysis of discrete variables was performed using the chi-squared test. Comparisons of continuous variables were performed either by a two-sided Student’s *t* test for unpaired samples or by one-way analysis of variance (ANOVA) with a Bonferroni correction applied where appropriate. Kaplan-Meier survival curves with log-rank tests were used for the analysis of the patient survival. Data for patients who were lost to follow-up were censored at the time of the last contact. Univariate and multivariate Cox proportional hazards models were used for estimation of hazard ratios (HR) and associated 95 % confidence intervals (CI). A *p* < 0.05 was considered as statistically significant.

## Results

One hundred four patients were enrolled in the study (86 males, 18 females; mean age at MPI after HTx 52.9 ± 13.1 years, mean time after HTx 3.6 ± 2.9 years). Patients’ characteristics are shown in Table 1. The mean follow-up time was 9.4 ± 3.1 years after MPI (median, 10.0 years; range, 2.2–13.7 years).

**Table 1:** Patients’ characteristics

Variable	All (*n* = 104)	Homogeneous perfusion (*n* = 79)	Inhomogeneous perfusion (*n* = 25)	*p* value
Age at date of HTx (years)	49.4 ± 12.6	49.4 ± 12.6	49.4 ± 13.3	0.98
Gender (men/women)	86 (83 %)/18 (17 %)	65 (82 %)/14 (18 %)	21 (84 %)/4 (16 %)	0.46
BMI	25.8 ± 3.3	25.9 ± 3.5	25.3 ± 2.8	0.51
Reason for HTx				
CAD	50 (48 %)	37 (47 %)	13 (52 %)	0.68
Dilated cardiomyopathy	44 (42 %)	35 (44 %)	9 (36 %)	0.07
Others	10 (10 %)	7 (9 %)	3 (12 %)	0.14
Systemic hypertension	88 (85 %)	65 (82 %)	23 (92 %)	0.78
Diabetes mellitus	18 (17 %)	14 (18 %)	4 (16 %)	0.68
Hypercholesterolemia	76 (73 %)	58 (73 %)	18 (72 %)	0.25
Renal failure	71 (68 %)	51 (65 %)	20 (80 %)	0.22
Malignancy	14 (13 %)	13 (16 %)	1 (4 %)	0.12
Peripheral artery disease	12 (12 %)	10 (13 %)	2 (8 %)	0.55
Beta blockers	26 (25 %)	19 (24 %)	7 (28 %)	0.55
Calcium channel antagonists	88 (85 %)	72 (91 %)	16 (64 %)	0.04
ACE inhibitors	27 (26 %)	19 (24 %)	8 (32 %)	0.39
Statins	94 (90 %)	73 (92 %)	21 (84 %)	0.77
Coronary interventions in the follow-up	15 (15 %)	13 (16 %)	2 (8 %)	0.06
MACE (incl. interventions)	20 (19 %)	15 (19 %)	5 (20 %)	0.14
Deaths in observation period	11 (11 %)	7 (9 %)	4 (16 %)	0.26

Values for age and BMI (body mass index) are expressed as mean ± standard deviation. All other data are absolute values

### Inhomogeneity

The degree of inhomogeneity was more pronounced in the stress perfusion (Table 2). There were no cases with inhomogeneous perfusion more pronounced in the rest study than in the stress study. Therefore, only the degree of inhomogeneity of the stress perfusion was further considered. Myocardial perfusion pattern was graded as homogeneous in 76 % of the patients (*n* = 79), while inhomogeneous myocardial perfusion was present in 24 % of the patients (*n* = 25), from which 19 SPECT scans were classified as moderately inhomogeneous and 6 as severely inhomogeneous.

**Table 2:** Frequency of inhomogeneous myocardial stress and rest perfusion

		Rest		
Stress	Inhomogeneity	No	Moderate	Severe
	No	79	0	0
	Moderate	0	19	0
	Severe	0	4	2
	Total	79	23	2

Concerning the prevalence of inhomogeneous myocardial perfusion, there were no significant differences between men (*n* = 21) and women (*n* = 4; *p* = 0.69). The mean time after HTx at the time of imaging did not differ significantly between patients with homogeneous and inhomogeneous perfusion (3.7 ± 2.9 years versus 3.1 ± 3.0 years, *p* = 0.42).

In our cohort, the mean maximum predicted heart rate (MPHR) achieved was 82.5 ± 7.8 %. Of the patients, 77.9 % (*n* = 81) reached a MPHR ≥85 %. Otherwise, the exercise test was abandoned due to symptoms (angina pectoris or dyspnea).

Semiquantitative perfusion scores are presented in Table 3. There was no significant correlation between the distribution of SRS or SSS between patients with homogeneous or inhomogeneous myocardial perfusion (*p* = 0.324 and *p* = 0.095, respectively).

**Table 3:** Summed rest scores (SRS) and summed stress scores (SSS)

		Inhomogeneity		Total
		No	Yes	
SRS	0	40	10	50
	1	20	9	29
	2	18	6	24
	3	1	0	1
Total		79	25	104
SSS	0	31	8	39
	1	21	6	27
	2	22	8	30
	3	5	3	8
Total		79	25	104

The presence of significant coronary artery stenosis was excluded in every patient. Of the 104 patients, 92 (88.5 %) had no detectable angiographic lesion, whereas 12 patients (11.5 %) presented with single vessel stenosis <50 %. Here, there were no statistical differences between the prevalence in the homogeneous and the inhomogeneous group.

Inhomogeneous myocardial perfusion did not correlate with the severity and frequency of previous cardiac allograft rejections (*p* = 0.71; not shown) and did not correlate with cardiac disease before transplantation (*p* = 0.88; not shown).

### LV function and follow-up

**Fig. 2:**
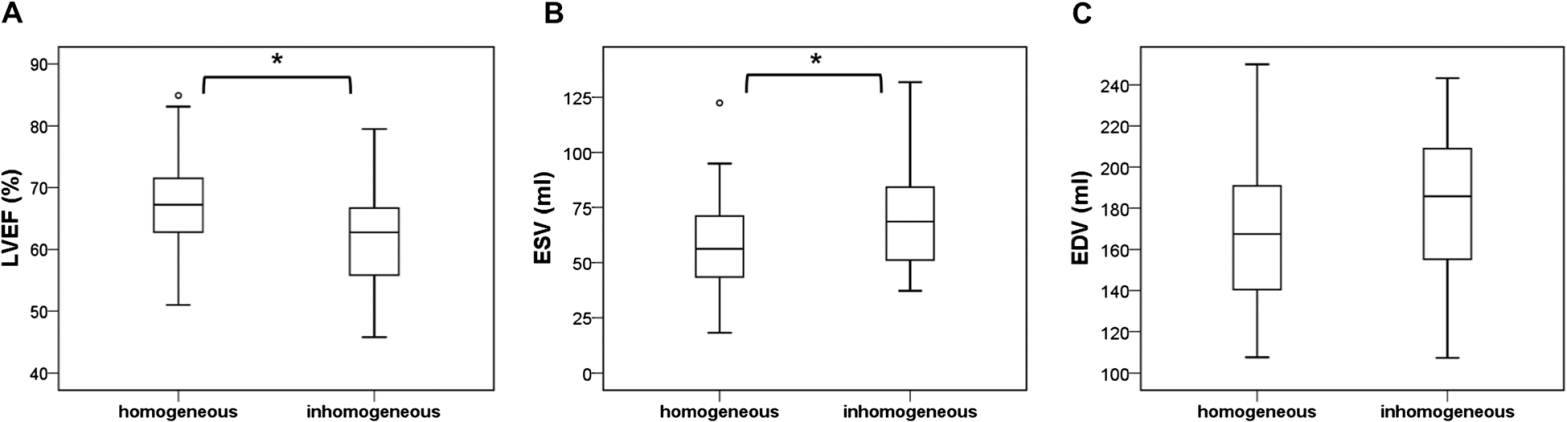
Comparison of LVEF (**a**), ESV (**b**), and EDV (**c**), assessed by gated SPECT. *Asterisk* indicates a *p* value <0.01

At the time of gated SPECT, LVEF was predominantly preserved but significantly lower in patients with inhomogeneous perfusion than in patients with homogeneous perfusion (61.9 ± 9.2 versus 67.7 ± 7.5 %; *p* = 0.008) and ESV was higher (70 ± 24 versus 54 ± 18 ml; *p* = 0.002; Fig. 2a, b). EDV did not differ significantly (177 ± 37 versus 165 ± 30 ml; *p* = 0.11; Fig. 2c).

Although statistically not significant, data showed a tendency of LVEF worsening with the degree of inhomogeneity: 67 ± 8 % in patients with homogeneous perfusion, 63 ± 9 % in patients with moderately inhomogeneous perfusion, and 59 ± 10 % in patients with severely inhomogeneous perfusion (*p* = 1.0, *p* = 0.29, and *p* = 0.11).

**Fig. 3:**
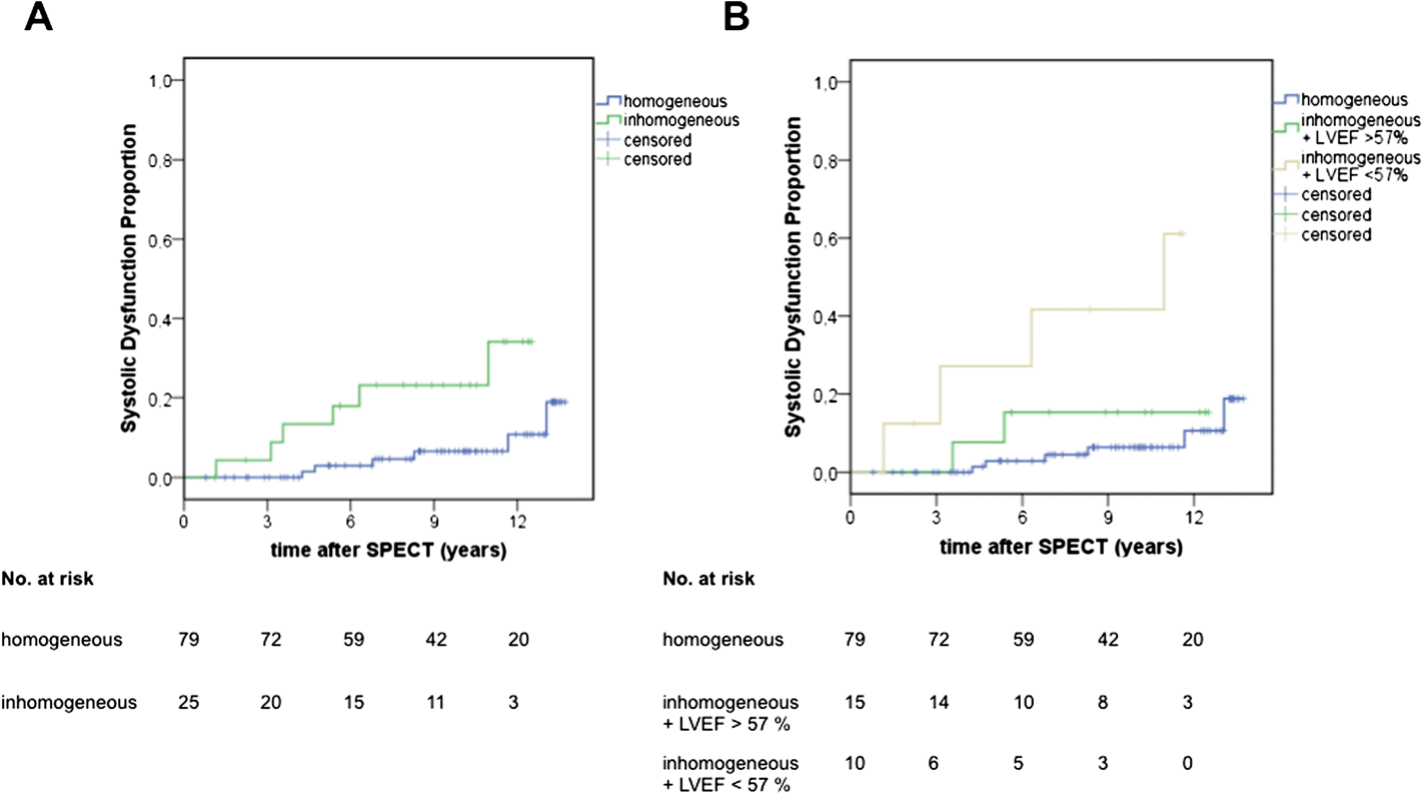
Cumulative incidence of allograft dysfunction. Comparison between patients with homogeneous and inhomogeneous perfusion (**a**) and more differentiated in patients with inhomogeneous perfusion and LVEF > versus <57 % (**b**)

In the follow-up, inhomogeneous myocardial perfusion was associated with a significantly more frequent decrease of LVEF <45 % (Fig. 3a). The univariate HR was 5.0 (95 % CI, 1.52–16.4; *p* < 0.01; Table 4). Particularly, the combined finding of inhomogeneous perfusion and LVEF ≤57 % (=threshold value calculated from control group data) in gated SPECT was associated with an increased risk for the development of allograft dysfunction (HR 3.5 [CI 1.74–6.98], *p* < 0.01; Fig. 3b).

**Table 4:** Univariate and multivariate analysis of potential risk factors with regards to LV dysfunction (first row) and MACE (second row)

		Univariate analysis			Multivariate analysis		
Variable		HR	95 % CI	*p* value	HR	95 % CI	*p* value
Perfusion inhomogeneity	No	Reference			Reference		
	Yes	5.0	1.52–16.4	0.008	5.59	1.69–18.5	0.005
		3.79	0.53–26.91	0.183			
ACR	≤grade 2R	Reference			Reference		
	≥grade 2R	0.18	0.04–0.81	0.025	0.16	0.34–0.73	0.018
		0.27	0.03–2.57	0.253			
Hypertension	No	Reference					
	Yes	0.39	0.12–1.29	0.123			
		0.54	0.06–5.17	0.537			
PAD	No	Reference					
	Yes	1.82	0.39–8.44	0.439			
		2.2	0.44–11.07	0.338			
Renal failure	No	Reference					
	Yes	2.96	0.64–13.57	0.164			
		1.74	0.180–16.87	0.632			
Diabetes	No	Reference					
	Yes	1.15	0.31–4.23	0.837			
		0.61	0.14–2.61	0.51			

In a multivariate analysis of several risk factors, only inhomogeneous myocardial perfusion was predictive for the development of allograft dysfunction (Table 4).

Previous cardiac allograft rejections ≥grade 2R (ISHLT 2004) were not predictive of the development of allograft dysfunction but in contrast associated with a preserved LV function (Table 4).

**Fig. 4:**
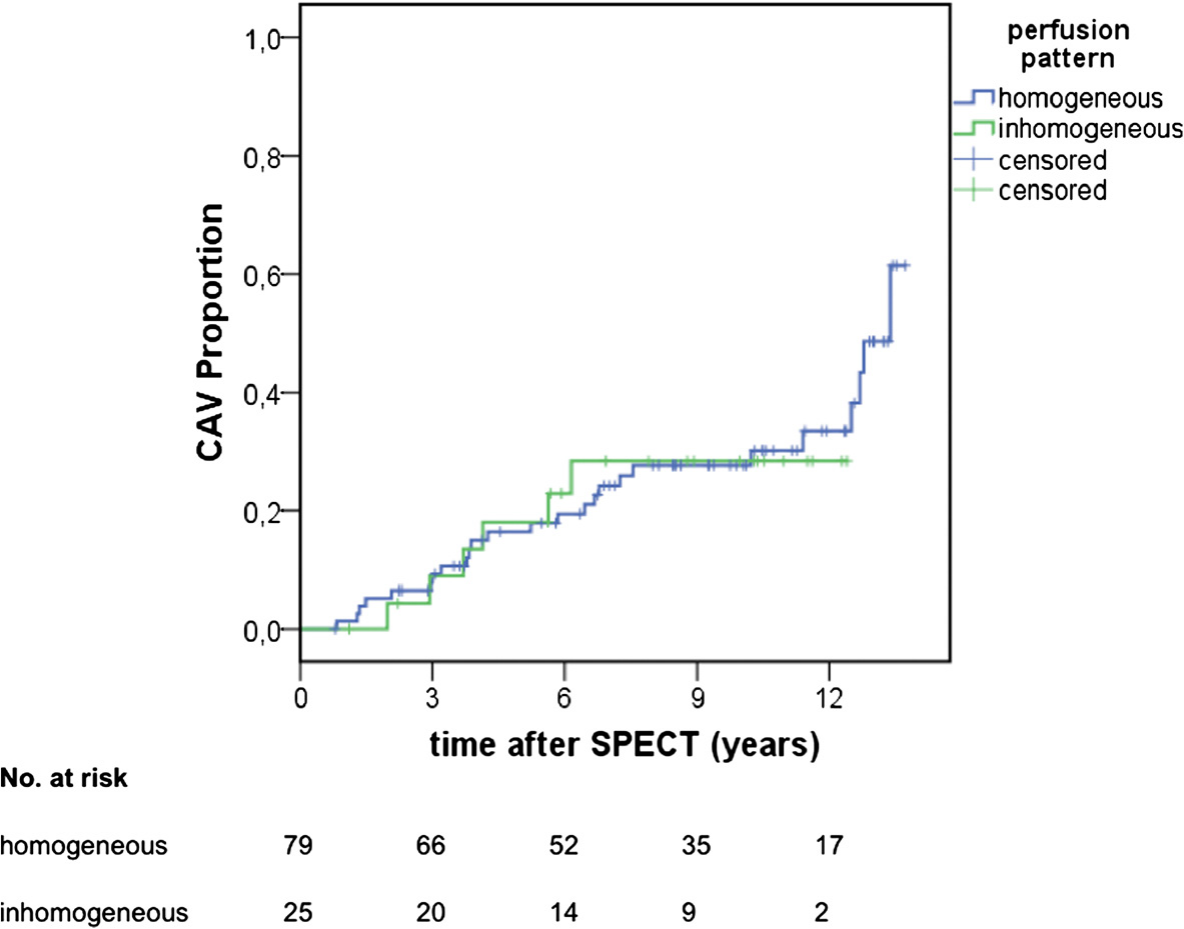
Cumulative incidence of epicardial CAV

**Fig. 5:**
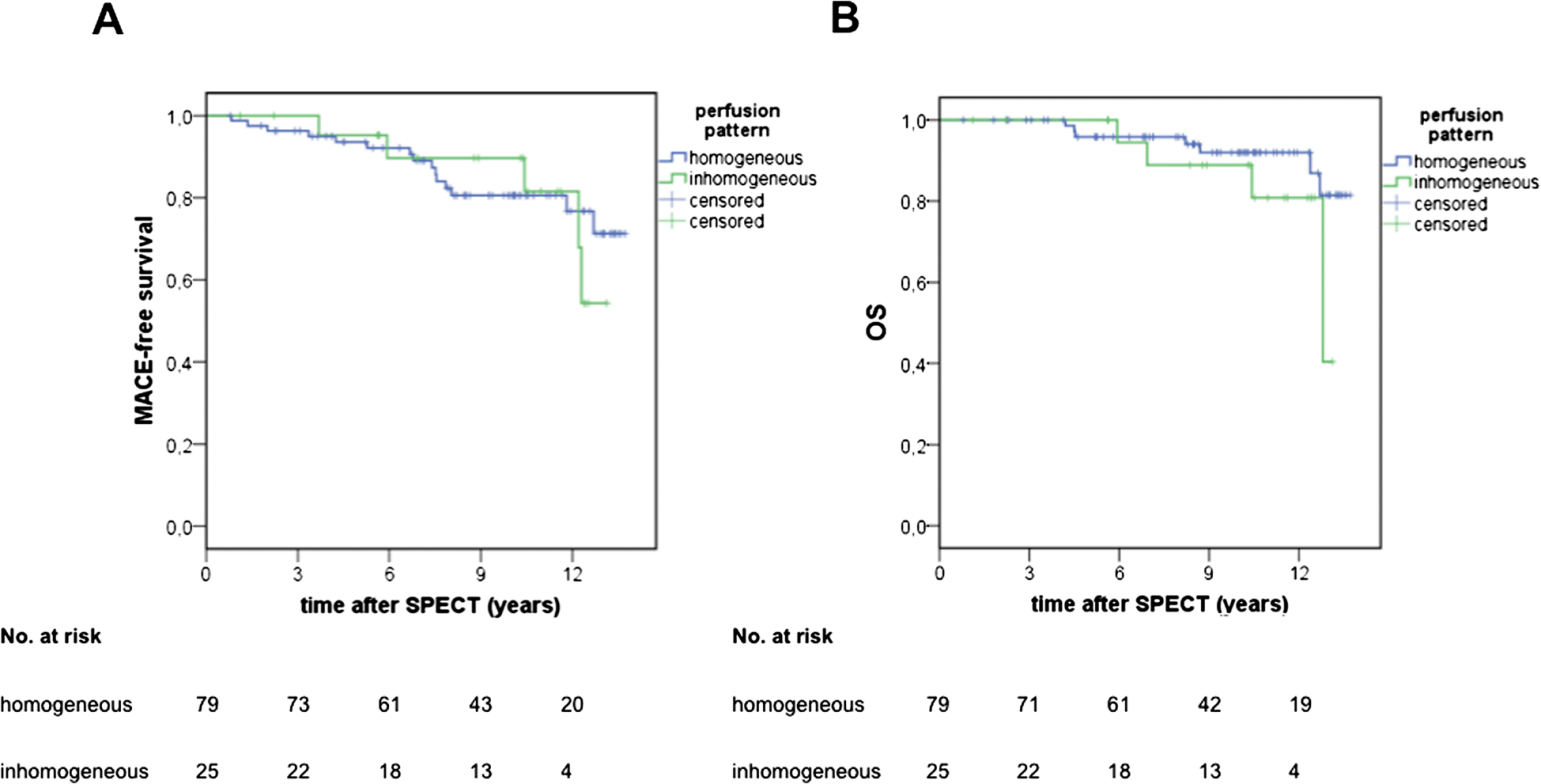
Cumulative incidence of MACE-free survival (**a**) and overall survival, OS (**b**)

No association was found between inhomogeneous myocardial perfusion and the manifestation of epicardial CAV in coronary angiography in the follow-up (Fig. 4). Moreover, no significant differences were found between the groups with regard to the occurrence of MACE or death of any cause in the follow-up period (Fig. 5a, b).

### Immunosuppression

Of the patients, 87.5 % (*n* = 91) initially received a cyclosporine-based immunosuppressive therapy, whereas 12.5 % of the patients received either everolimus (*n* = 1), sirolimus (*n* = 2), or tacrolimus (*n* = 10). In the follow-up, 36.5 % (*n* = 38) of the patients received a change in immunosuppression, whereas in 26 % (*n* = 27) of the cases, a change towards everolimus was performed. This change was more frequently performed in the group with initially inhomogeneous myocardial perfusion pattern (36 versus 23 %).

## Discussion

In the follow-up of heart transplantation, inhomogeneous perfusion is a frequent finding in myocardial perfusion gated SPECT. However, its clinical significance is still uncertain. Only a few number of published reports have analyzed myocardial perfusion inhomogeneity in ^201^Thallium myocardial SPECT of heart transplant recipients so far [13, 15]. Here, the frequency and extent of perfusion inhomogeneity was reported to increase with time after HTx. However, this finding did not correlate with the presence of allograft vasculopathy as detected by coronary angiography and IVUS [15]. Thus, perfusion inhomogeneity in SPECT was assumed to be caused by small vessel alterations. Despite these first important findings, the few listed studies were either correlative or only covered a rather short follow-up time.

In this study, HTx patients had a median follow-up of ~10 years after a first myocardial perfusion gated SPECT in the course of heart transplantation. At the time of SPECT imaging, patients with inhomogeneous perfusion already had a preserved but significantly lower LVEF in comparison to patients presenting with homogeneous myocardial perfusion, with a non-significant trend of further deterioration of LVEF with a higher degree of inhomogeneity. In the follow-up, patients with inhomogeneous myocardial perfusion developed left ventricular allograft dysfunction more frequently than patients with homogeneous myocardial perfusion. Among these patients, particularly those showing perfusion inhomogeneity in combination with an already slightly restricted LVEF in gated SPECT were at a higher risk for the development of cardiac allograft dysfunction than patients with inhomogeneous perfusion but preserved LVEF. In a multivariate analysis including several risk factors, only inhomogeneous myocardial perfusion turned out as an independent predictor for the development of allograft dysfunction. Surprisingly, former acute allograft rejections were associated with a preserved LV function in the follow-up. This finding is confounding at first sight but may be explained by a possibly more aggressive immunosuppressive therapy in these individuals.

In non-transplanted patients, MPHR ≥ 85 % is targeted in a physical stress test. Although MPHR is not established in the (denervated) HTx population and therefore may only serve as an approximate parameter for cardiac stress in HTx patients, the targeted MPHR was virtually achieved in our cohort. Thus, the diagnostic accuracy of MPI should not be limited. In line with results from former studies, perfusion inhomogeneity did not correlate with the presence of epicardial CAV in coronary angiography. On the other hand, the degree inhomogeneity was more pronounced in the stress perfusion. Thus, the results of our study support the hypothesis of microvascular disease leading to inhomogeneous myocardial perfusion in HTx patients. Microvasculopathy is consecutively causing a diffuse and chronic ischemic cardiomyopathy manifested by progressive left ventricular dysfunction and most probably fibrosis [20]. Since no association between the SPECT perfusion pattern and the frequency or severity of prior allograft rejections could be observed, a diffuse myocardial damage due to former acute allograft rejections is unlikely to cause the observed perfusion inhomogeneity.

Inhomogeneous myocardial perfusion in SPECT can also be observed in non-transplanted patients with angiographically normal coronary arteries. Formerly often claimed as false positive, studies suggest that endothelial dysfunction might be the reason for the observed perfusion patterns in these patients [21]. IVUS measurements revealed that defects in myocardial perfusion scintigraphy correlate with occult atherosclerosis without the presence of significant epicardial coronary artery stenosis in angiography [22]. These results suggest that regionally impaired myocardial perfusion reserve due to microvascular dysfunction may cause relative hypoperfusion in myocardial perfusion gated SPECT. Using myocardial perfusion positron emission computed tomography (PET), quantitative perfusion abnormalities have been verified in patients with arterial hypertension or patients with dilated and hypertrophic cardiomyopathy without significant epicardial coronary artery stenosis in coronary angiography, respectively—findings which underline the importance of coronary microcirculation with regard to analysis of myocardial perfusion images [23]. Myocardial perfusion is also often impaired in diabetic patients [24], and accumulating evidence suggests that small vessel disease also plays a significant role in the development of cardiac dysfunction and the development of ‘diabetic cardiomyopathy’ [25]. That kind of cardiomyopathy is characterized by myocardial dysfunction in the absence of coronary artery disease [26]. Most probably, microvascular dysfunction is also responsible for alterations in heart function such as increased ventricular wall stiffness and reduced cardiac contractility [27]. Similar to the findings in these cardiomyopathies, HTx patients enrolled in our study were free of epicardial CAV or coronary artery disease although the pathophysiological processes leading to CAV basically differ from those causing diabetic microangiopathy. Nevertheless, microvasculopathy which is due to medial rather than endothelial disease or a combination of both [4] is most probably responsible for the more frequent development of cardiac allograft systolic dysfunction in HTx patients presenting with inhomogeneous perfusion in SPECT imaging. Although the inhomogeneous perfusion pattern was not associated with impaired survival of the patients in our follow-up period (≈10 years after SPECT), it has been shown that microvasculopathy is associated with a worse overall survival in the long-term follow-up (up to 15 years after HTx) [4]. Recently, McArdle et al. could demonstrate that quantitation of coronary flow reserve by dynamic PET has an additional prognostic value in HTx patients. They could show that patients with impaired coronary flow reserve were at higher risk for adverse events in a median follow-up of about 18 months [28]. Particularly, the analysis of coronary flow reserve in addition to the analysis of relative perfusion differences, which may miss microvascular disease, enhanced the prognostic power of myocardial perfusion imaging. In this context, further prospective studies using quantitative myocardial perfusion PET may shed light on the hypothesis of microvasculopathy causing inhomogeneity beyond the SPECT findings presented here.

Most importantly, HTx patients presenting with inhomogeneous myocardial perfusion are at an increased risk for the development of future allograft dysfunction. Therefore, the analysis of inhomogeneity of myocardial perfusion and the integration of this finding into the clinical report can aid the management of HTx patients in the follow-up of transplantation, although patients may not initially present with significant myocardial ischemia, epicardial CAV, or signs of heart failure. The presence of inhomogeneous perfusion could alert clinicians early to consider a more intensive monitoring of left ventricular function and potential additional treatments in order to prevent a further progression of microvasculopathy and the development of progressive allograft failure. Interestingly, despite this finding, there were no statistically significant differences between patients with homogeneous and inhomogeneous perfusion with regard to the occurrences of MACE or deaths. The overall survival is generally good in the cohort, independent from the extent of perfusion inhomogeneity. A possible explanation could be the more frequent change to everolimus in the inhomogeneous group which may have reduced event rates. Due to the clinical setting of the study, clinicians were aware of the imaging results. However, changes of immunosuppressive therapy were not made depending on the grade of inhomogeneity reported.

### Limitations

A limitation is given by the mixed cohort and the retrospective design of the study—that is not every patient could be included from the date of HTx on, and the recruitment period was long. Thus, we do not know if patients presenting with inhomogeneous perfusion in the first SPECT studies available may have had presented with similar perfusion patterns already at earlier time points. On the other hand, the results should be comparable, since there is no difference in the mean time interval after HTx between the analyzed groups. Moreover, the presented results may be less applicable to women since the cohort was dominated by male individuals.

A methodological limitation may be the grading system for inhomogeneous myocardial perfusion pattern. The cutoff values used for grading of inhomogeneity were defined in comparison to visual impression of myocardial perfusion pattern. Although the intra- and interobserver agreement was very good, the grading system used is obviously user-dependent and subjective by nature. Nevertheless, we think that that kind of grading of myocardial perfusion inhomogeneity is reasonably feasible, apart from that there is a lack of custom-made software for quantitation of perfusion inhomogeneity so far. A formerly published score of inhomogeneity [15] was not deemed suitable for the analysis, since this method was based on ^201^Thallium SPECT images without attenuation correction, and the score excluded the whole inferior left ventricular wall.

Another limitation of the study is the lack of quantitative data like absolute blood flow and cardiac flow reserve which could confirm the hypothesis of microvascular dysfunction better than SPECT can do.

## Conclusions

In summary, inhomogeneous myocardial stress perfusion in gated SPECT of HTx patients is associated with a poor prognosis concerning the development of allograft dysfunction, defined as LVEF <45 %. On the other hand, inhomogeneity was neither associated with the future development of epicardial CAV nor with the occurrence of MACE or with all cause mortality.

## Abbreviations

ASNC: American Society of Nuclear Cardiology
BMI: body mass index
CAD: coronary artery disease
CAV: cardiac allograft vasculopathy
EDV: enddiastolic volume
ESV: endsystolic volume
HR: hazard ratio
HTx: heart transplantation
LVEF: left ventricular ejection fraction
MACE: major cardiac event
MPHR: maximum predicted heart rate
MPI: myocardial perfusion imaging
MBq: megabecquerel
OS: overall survival
PET: positron emission computed tomography
SDS: summed difference score
SPECT: single photon emission computed tomography
SRS: summed rest score
SSS: summed stress score
TTE: transthoracic echocardiogram
